# Multisystem screening reveals SARS‐CoV‐2 in neurons of the myenteric plexus and in megakaryocytes

**DOI:** 10.1002/path.5878

**Published:** 2022-03-31

**Authors:** Sandra Gray‐Rodriguez, Melanie P Jensen, Maria Otero‐Jimenez, Brian Hanley, Olivia C Swann, Patrick A Ward, Francisco J Salguero, Nadira Querido, Ildiko Farkas, Elisavet Velentza‐Almpani, Justin Weir, Wendy S Barclay, Miles W Carroll, Zane Jaunmuktane, Sebastian Brandner, Ute Pohl, Kieren Allinson, Maria Thom, Claire Troakes, Safa Al‐Sarraj, Magdalena Sastre, Djordje Gveric, Steve Gentleman, Candice Roufosse, Michael Osborn, Javier Alegre‐Abarrategui

**Affiliations:** ^1^ Department of Brain Sciences, Imperial College London Hammersmith Hospital London UK; ^2^ Department of Cellular Pathology, Northwest London Pathology Charing Cross Hospital Campus London UK; ^3^ Department of Immunology and Inflammation Imperial College London London UK; ^4^ Department of Infectious Disease Imperial College London London UK; ^5^ Chelsea and Westminster NHS Foundation Trust London UK; ^6^ National Infection Service, United Kingdom Health Security Agency Salisbury UK; ^7^ Pandemic Sciences Centre, Nuffield Department of Medicine Oxford University Oxford UK; ^8^ Department of Neuropathology UCL Queen Square Institute of Neurology London UK; ^9^ Department of Cellular Pathology Queen Elizabeth Hospital Birmingham/University Hospitals Birmingham Birmingham UK; ^10^ Department of Neuropathology Cambridge University Hospitals NHS Foundation Trust Cambridge UK; ^11^ Basic and Clinical Neuroscience Department, Institute of Psychiatry Psychology & Neuroscience, King's College London London UK; ^12^ Multiple Sclerosis and Parkinson's Tissue Bank, Imperial College London Hammersmith Hospital London UK

**Keywords:** COVID‐19, SARS‐CoV‐2, myenteric plexus, megakaryocytes, immunohistochemistry, gastrointestinal tract, Parkinson's Disease, neurons, tropism, enteric nervous system

## Abstract

SARS‐CoV‐2, the causative agent of COVID‐19, typically manifests as a respiratory illness, although extrapulmonary involvement, such as in the gastrointestinal tract and nervous system, as well as frequent thrombotic events, are increasingly recognised. How this maps onto SARS‐CoV‐2 organ tropism at the histological level, however, remains unclear. Here, we perform a comprehensive validation of a monoclonal antibody against the SARS‐CoV‐2 nucleocapsid protein (NP) followed by systematic multisystem organ immunohistochemistry analysis of the viral cellular tropism in tissue from 36 patients, 16 postmortem cases and 16 biopsies with polymerase chain reaction (PCR)‐confirmed SARS‐CoV‐2 status from the peaks of the pandemic in 2020 and four pre‐COVID postmortem controls. SARS‐CoV‐2 anti‐NP staining in the postmortem cases revealed broad multiorgan involvement of the respiratory, digestive, haematopoietic, genitourinary and nervous systems, with a typical pattern of staining characterised by punctate paranuclear and apical cytoplasmic labelling. The average time from symptom onset to time of death was shorter in positively versus negatively stained postmortem cases (mean = 10.3 days versus mean = 20.3 days, *p* = 0.0416, with no cases showing definitive staining if the interval exceeded 15 days). One striking finding was the widespread presence of SARS‐CoV‐2 NP in neurons of the myenteric plexus, a site of high ACE2 expression, the entry receptor for SARS‐CoV‐2, and one of the earliest affected cells in Parkinson's disease. In the bone marrow, we observed viral SARS‐CoV‐2 NP within megakaryocytes, key cells in platelet production and thrombus formation. In 15 tracheal biopsies performed in patients requiring ventilation, there was a near complete concordance between immunohistochemistry and PCR swab results. Going forward, our findings have relevance to correlating clinical symptoms with the organ tropism of SARS‐CoV‐2 in contemporary cases as well as providing insights into potential long‐term complications of COVID‐19. © 2022 The Authors. *The Journal of Pathology* published by John Wiley & Sons Ltd on behalf of The Pathological Society of Great Britain and Ireland.

## Introduction

SARS‐CoV‐2 is an enveloped RNA virus, containing an outer lipid membrane derived from the host cell. The viral envelope contains the spike (S) protein that binds to target cell receptors—most notably the ACE2 receptor, which is expressed in multiple tissues. Entry is followed by cleavage of the S protein by proteases such as TMPRSS2, leading to fusion of viral and cellular membranes [1]. TMPRSS2 is expressed in a range of tissues, although TMPRSS2 and ACE2 are coexpressed only in a subset of cells; for example, respiratory epithelial cells, enterocytes, and neurons of the myenteric plexus [1, 2]. Additional structural viral proteins include the envelope (E), membrane (M), and nucleocapsid (N) proteins, the latter of which encapsulates the viral RNA. Molecular detection of such proteins and/or viral RNA, via immunohistochemistry and *in situ* hybridisation, respectively, is emerging as a method whereby the tissue tropism of SARS‐COV‐2 can be elucidated [3].

Coronavirus disease 2019 (COVID‐19), the clinical disease caused by SARS‐CoV‐2, primarily affects the respiratory tract, although there is increasing evidence suggesting involvement of multiple organ systems. Clinically, a subset of patients present with alternative symptoms including gastrointestinal; for example, diarrhoea and nausea [4, 5, 6]. Reported neurological complications following SARS‐CoV‐2 infection include anosmia, ageusia, headache, impaired consciousness, and cognitive deficits [7]. Moreover, it appears that respiratory failure is not the sole cause of death in COVID‐19; thrombosis is emerging as an important cause of morbidity and mortality, particularly in patients with underlying cardiovascular conditions [6]. This is thought to relate to endothelial involvement, given the expression of ACE2 receptor on these cells [4, 5, 6].

Several histopathological studies have been performed on postmortem tissue from COVID‐19 patients, including autopsies of patients who died of severe SARS‐CoV‐2 infection. Common histological alterations in these patients include diffuse alveolar damage (DAD), thrombotic events, and immune cell depletion [8]. The clinicopathological correlation for several organs potentially affected in COVID‐19, such as the kidney, pancreas, and liver are, however, not fully understood. Special attention has been paid to the nervous system in the search for pathological alterations responsible for the neurological symptoms of COVID‐19 given the neuroinvasive potential of SARS‐CoV‐2 [9]. Potential long‐term neuropathological sequelae of COVID‐19 are also of interest, as there is accumulating evidence that environmental agents including viruses may play a role in the initiation of the most common neurodegenerative diseases [10, 11, 12, 13]. Histopathological investigations of fatal COVID‐19 patients with neurological syndromes have so far not shown definitive evidence of viral protein or RNA in the brain, despite some studies reporting neuropathological abnormalities such as microglial activation or focal lymphocytic infiltrates [14, 15, 16, 17]. This suggests that neuroinvasion might not be responsible for neurological symptoms, which could instead be due to inflammatory responses or critical care treatment. Outside of the brain, the enteric nervous system (ENS) has been proposed as an entry point for SARS‐CoV‐2 into the central nervous system (CNS) *via* the vagus nerve reaching the medulla, given the expression of the key viral entry receptors (ACE2 and TMPRSS2) in neurons of the myenteric plexus [2].

In this study, we characterise the systemic tropism of SARS‐CoV‐2 in a cohort of fatal COVID‐19 cases as well as in antemortem brain and tracheal biopsies using validated immunohistochemistry against SARS‐CoV‐2 nucleocapsid protein (NP), a structural protein critical for packaging of the viral genome [18]. We find that overall, the presence of SARS‐CoV‐2 NP matches the cell types with known high ACE2 receptor expression, and we describe novel extrapulmonary findings that implicate peripheral nervous system involvement (more specifically, the ENS) as well as digestive, genitourinary, and haematopoietic systems.

## Methods

### Cohort selection

We obained tissue from 36 patients to study the cellular tropism of SARS‐CoV‐2 by immunohistochemistry. The samples comprised tissue from 16 postmortem examinations and 16 antemortem biopsy specimens from the peaks of the pandemic in 2020 and four pre‐COVID postmortem controls (supplementary material, Table S1). Of the 16 postmortem cases from 2020 (PM1‐PM16, Table 1), 10 had multisystem organ sampling available (nine *via* full‐body autopsies and one *via* percutaneous biopsy sampling), whilst only brain tissue was available for six. All 16 postmortem cases had SARS‐CoV‐2 infection confirmed by PCR testing for SARS‐CoV‐2 RNA in nasopharyngeal swabs antemortem, and clinical courses consistent with COVID‐19. Details of patient selection for 14 of the cases have been previously described (PM 1–10 [8], PM 11–12 [14], PM 15–16 [19]).

**Table 1:** Clinical characteristics of COVID‐19 cases.

Case	Age (years)	Sex	Comorbidities	Clinical course	Symptom onset to time of death or biopsy (days)	Postmortem interval (days)	Cause of death after autopsy
PM1	61	Male	COPD, ischaemic heart disease	Respiratory arrest with ambulance services. Cardiac arrest upon presentation to the emergency department	10	5	1a Diffuse alveolar damage and myocardial infarction 1b SARS‐CoV‐2 infection and coronary artery atherosclerosis (stented) 2 Ischaemic heart disease, liver cirrhosis, elevated BMI
PM2	64	Male	Obstructive sleep apnoea, benign prostatic hyperplasia, migraine, umbilical hernia repair	Admitted from an external hospital after 3 days of intubation and ventilation for COVID‐19 pneumonitis. Worsening oxygen requirements and haemodynamic instability	13	9	1a Diffuse alveolar damage 1b SARS‐COV‐2 infection 2 Obstructive sleep apnoea
PM3	69	Female	COPD, obstructive sleep apnoea, cor pulmonale, ischaemic heart disease, hypertension, type 2 diabetes mellitus, peripheral neuropathy	Admitted with symptoms of COVID‐19 pneumonitis and diarrhoea. Developed acute kidney injury and deteriorated with tachypnoea and tiring. Referred for palliation and treatment not escalated above ward level	8	7	1a Pulmonary oedema and diffuse alveolar damage 1b SARS‐CoV‐2 infection 2 Obesity, hypertension, type II diabetes mellitus, smoker, ischaemic heart disease, obstructive sleep apnoea
PM4	78	Male	Dementia, hypertension, type 2 diabetes mellitus, osteoarthritis	Admitted with symptoms of COVID‐19 pneumonitis. Clinically deteriorated with desaturations. Referred for palliation and treatment not escalated above ward level	12	6	1a Diffuse alveolar damage and haemophagocytosis 1b SARS‐CoV‐2 infection 2 Dementia, frailty, hypertension
PM5	22	Male	Obesity, hypothyroidism	Admitted from an external hospital with suspected COVID‐19 pneumonia and a subacute right middle cerebral artery (MCA) infarct. Intubated and ventilated. CT head showed a haemorrhagic transformation into the right MCA infarct. Developed multiorgan failure and did not respond to maximal medical treatment	27	4	1a Multiorgan failure 1b Disseminated Mucormycosis 1c SARS‐CoV‐2 infection 2 Elevated body mass index, hypothyroidism, steatohepatitis
PM6	24	Male	Non‐alcoholic steatohepatitis, lichen planus, gonadotrophin releasing hormone deficiency	Admitted with symptoms of COVID‐19 pneumonitis and underwent an out of hospital cardiac arrest. Transferred to intensive care but died shortly after with worsening respiratory failure	8	9	1a Diffuse alveolar damage 1b SARS‐CoV‐2 infection
PM7	79	Male	Hypercholesterolaemia, trigeminal neuralgia	Admitted with symptoms of COVID‐19 pneumonitis. Admitted to intensive care unit due to worsening hypoxia. Deteriorated with escalating inotropic requirements, worsening acute kidney disease, coagulopathy and rising inflammatory markers	23	6	1a Diffuse alveolar damage 1b SARS‐CoV‐2 infection 2 Left ventricular hypertrophy, coronary atherosclerosis
PM8	97	Male	Recurrent urinary tract infections, dementia, bladder cancer, anaemia, hypothyroidism, glaucoma, alcohol‐related liver disease, pacemaker in‐situ	Admitted with general malaise and initially treated as a UTI. During his admission developed fever, lethargy and cough. Began to require supplemental oxygen and CRP began to rise. Referred for palliation and treatment not escalated above ward level. Chest x‐ray showed worsening bilateral consolidation and he developed worsening respiratory failure	23	6	1a Diffuse alveolar damage and widespread thrombosis 1b SARS‐CoV‐2 infection 2 Hepatic fibrosis, dementia, frailty and cardiac amyloidosis
PM9	79	Female	Chronic obstructive pulmonary disease, lupus erythematosus, hypertension, type 2 diabetes mellitus, vitamin B12 deficiency	Admitted with diarrhoea and vomiting. PCR test for clostridium difficile was positive and she was treated with antibiotics. During her admission she had ongoing elevated inflammatory markers and began to desaturate on room air. She developed worsening respiratory failure. Referred for palliation and treatment not escalated above ward level	24	7	1a Diffuse alveolar damage 1b SARS‐CoV‐2 infection 2 Hypertension, diabetes mellitus type II, chronic obstructive pulmonary disease
PM10	77	Female	Hypertension, left ventricular hypertrophy, osteoporosis, osteoarthritis, chronic kidney disease, vasculitis, hypercholesterolaemia, hiatus hernia, vitamin B12 and D deficiency, gallstone pancreatitis	Admitted with COVID‐19 pneumonitis, abdominal pain and diarrhoea. Developed worsening tachypnoea and hypoxia. Referred for palliation and treatment not escalated above ward level	15	1	1a Diffuse alveolar damage 1b SARS‐CoV‐2 infection 2 Hypertension, chronic kidney disease
PM11	71	Male	Nil	Admitted with COVID‐19 pneumonitis. Transferred to intensive care due to worsening respiratory failure. Failed weaning from mechanical ventilation with persistent hypercapnia. CT head revealed diffuse bilateral gyral calcification. His neurological status remained poor off sedation	46	9	1a COVID‐19 pneumonitis
PM12	66	Male	Ischaemic and hypertensive heart disease, type 2 diabetes mellitus, COPD	Admitted with COVID‐19 pneumonitis. Transferred to critical care where he remained in multiorgan failure despite prolonged ventilation and continuous renal replacement therapy. Slow to wake from sedation. MRI brain revealed nil acute lesions. Due to worsening gas exchange, rising inotropic requirements and limited neurological improvement he was switched to supportive care	>30	9	1a Multiorgan system failure 1b COVID‐19 infection 2 Ischaemic and hypertensive heart disease, type 2 diabetes, chronic obstructive pulmonary disease
PM13	55	Male	Nil	Admitted and treated for COVID‐19 pneumonia, requiring intensive care. He deteriorated suddenly 1 week into his admission and CT scan showed a pulmonary embolism. He was thrombolysed and received extracorporeal membrane oxygenation. 9 days later his neurological function worsened and a CT head showed a catastrophic intracranial haemorrhage with generalised cerebral oedema. It was agreed this was not survivable and best supportive care was provided.	>19	15	1a Intracerebral haemorrhage 1b COVID‐19
PM14	76	Male	Rheumatoid arthritis, asthma, asbestos exposure	Admitted with COVID‐19 pneumonitis, requiring intensive care. Respiratory failure worsened 2 weeks into his admission and he was sedated and ventilated. He continued to worsen and died 1 month after admission.	29	15	1a COVID‐19 pneumonitis
PM15	52	Male	Type 2 diabetes mellitus, asthma	Admitted to hospital with COVID‐19 pneumonitis, requiring intubation and ventilation two days into admission. The patient developed multifocal cerebral and cerebellar infarcts, a saddle pulmonary embolism and acute kidney injury. Repeat imaging showed evolution of the multiple infarcts and the patient died shortly after.	20	Not known	1a Multiorgan failure 1b COVID‐19 and pulmonary embolism 2 Type 2 diabetes mellitus, asthma
PM16	66	Female	Hypertension, asthma	Admitted to hospital with COVID‐19 pneumonitis, requiring intubation and ventilation on hospitalisation and renal replacement therapy for acute kidney injury. The patient remained unresponsive, and MRI of the brain showed multiple disseminated small infarcts in the subcortical cerebral white matter and microbleeds. The patient died due to persistent multiorgan failure.	31	4	1a Multiorgan failure with cerebral infarcts 1b COVID‐19
BB1	59	Female	Aplastic anaemia, MGUS, treated breast cancer, hypertension, non‐alcoholic fatty liver disease, hypercholesterolaemia	Admitted to hospital with mild COVID‐19 pneumonitis and recurrent episodes of vacant staring and speech arrest associated with a generalised tonic–clonic seizure, followed by reduced consciousness, requiring ICU admission and intubation and ventilation. MRI brain showed features in keeping with a severe necrotising encephalitis.	20	Not applicable	Not applicable

Adapted from (PM1‐10) [8], (PM11‐12) [14], (PM15‐16) [19], and (BB1) [20]. Tissue for PM1‐16 was obtained postmortem and BB1 is an antemortem brain biopsy. Detailed case vignettes of PM1‐10 can be found within the supplementary material of Hanley *et al* [8].

Of the antemortem biopsy specimens, one was a brain biopsy (BB1, Table 1) and 15 were tracheal biopsies from patients admitted to intensive care during 2020 (TB1–TB15, Table 4). The patient corresponding to sample BB1 had a clinical presentation that was strongly consistent with COVID‐19 and has been described in detail elsewhere [20]. For the tracheal biopsies, tissue samples were obtained from tracheal windows excised at surgical tracheostomy, performed in critically ill mechanically ventilated patients as part of their routine intensive care unit (ICU) care. SARS‐CoV‐2 infection status was determined by reverse transcription polymerase chain reaction (RT‐PCR) or multiplex tandem PCR (MT‐PCR) of intravitam nasopharyngeal swab samples. Samples were obtained from the Imperial College Healthcare Tissue Bank (ICHTB). The ICHTB is approved by Wales REC3 to release human material for research (17/WA/0161), and the samples for this project (with approvals R20012 and R20027) were issued from subcollection reference number MED_MO_20_011. Additional tissue was obtained from the University College London Hospitals NHS Trust (ref 06–202021‐SE) and Brain UK (ref 20‐007). For PM1‐16, the next of kin gave their consent for tissues taken at coronial autopsy to be used for education, research, and audit.

### Immunohistochemistry (IHC)

Sections (4 μm) were prepared from formalin‐fixed, paraffin‐embedded tissue blocks. Following deparaffinisation and rehydration, slides were assembled for staining using Shandon Sequenza Immunostaining Center slide racks (Fisher Scientific, Thermo Fisher Scientific, Waltham, MA, USA). After 1 h blocking endogenous peroxidases with 1% hydrogen peroxide in PBS, heat‐induced epitope retrieval was performed using 0.1 m sodium citrate buffer (pH 6) for 20 min. For anti‐ChAT, antigen retrieval was performed with a 25‐min autoclave cycle, and for anti‐NOS1 by heating for 10 min in a 700 W microwave. Blocking of nonspecific binding was achieved using 10% normal horse serum (Vector Laboratories, Burlingame, CA, USA) diluted in PBS and sections were incubated overnight at 4 °C with mouse anti‐SARS‐CoV‐2 Nucleoprotein monoclonal antibody (1:500; Creative Diagnostics, Shirley, NY, USA; clone 4B21). Other primary antibodies used were rabbit anti‐integrin beta 3 monoclonal antibody (CD61, 1:1000; Abcam, Cambridge UK; ab227702), rabbit anti‐ACE2 monoclonal antibody (1:500; Abcam, ab15348), goat anti‐ChAT polyclonal antibody (1:50; MilliporeSigma, Burlington, MA, USA; AB144P), and mouse monoclonal anti‐NOS1 (nNOS, 1:50; Santa Cruz Biotechnology, Dallas TX, USA; sc‐5302). After washing three times with PBS, secondary antibodies were applied using the ImmPRESS horseradish peroxidase (HRP) horse or goat antimouse IgG polymer detection kit (Vector Laboratories). Following a further three PBS washes, staining was visualised using 3,3'‐diaminobenzidine (DAB) and hydrogen peroxide as part of the ImmPACT DAB substrate HRP kit (Vector Laboratories). Sections were then counterstained with Mayer's haematoxylin (MilliporeSigma), dehydrated, cleared and mounted with DPX mountant. When labelling for multiple antigen targets, the VECTASTAIN ABC‐AP Kit (Vector Laboratories), containing biotinylated anti‐rabbit secondary antibody, was used. Visualisation of staining was then achieved with HIGHDEF blue IHC chromogen (AP). Following this, slides were dehydrated, cleared, and mounted with EcoMount (Biocare Medical, Pacheo, CA, USA). Microscopic images were acquired with a DS‐Fi2 Digital Camera attached to a Nikon Eclipse 50i microscope using a DS‐U3 Digital Camera Controller and NIS‐Elements image acquisition software (Nikon, Melville, NY, USA). Image processing was performed using Adobe Photoshop CS2 (Adobe Systems, San José, CA, USA).

### Western blotting

Cell lysates derived from SARS‐CoV‐2‐infected Vero E6 cultured cells containing NuPAGE LDS Sample Buffer (Invitrogen, Thermo Fisher Scientific) and 2‐mercaptoethanol were boiled for 10 min and then loaded into a NuPAGE 4–12% Bis‐Tris gels (Invitrogen) and run with 1X NuPAGE MES Running Buffer at 120 V. Proteins were then transferred onto methanol‐activated PVDF membranes (400 mA, 1 h). Blocking nonspecific binding was accomplished using 5% semi‐skimmed milk in Tris‐buffered saline (TBS) with 0.2% Tween 20 (TBS‐T) for 1 h. After washing with TBS‐T three times, membranes were incubated with mouse anti‐SARS‐CoV‐2 Nucleoprotein monoclonal antibody (1:1000; Creative Diagnostics, clone 4B21) diluted in 1% bovine serum albumin (BSA) and 0.01% sodium azide in TBS‐T overnight at 4 °C. The membrane was subsequently washed again three times with TBS‐T and then incubated with HRP‐conjugated secondary mouse antibody (1:3000, Sigma‐Aldrich, AP308P) in 5% milk. After 1 h and three TBS‐T washes, HRP detection with Pierce ECL western blotting substrate (Thermo Fisher Scientific) allowed for visualisation with the GeneGnome XRQ chemiluminescent imager. The membrane was stripped (ReBlot Plus Strong Antibody Stripping Solution [10×], SigmaMillipore), blocked again with 5% semi‐skimmed milk in TBS‐T, and then incubated with anti‐beta actin primary antibody (1:25000; Abcam, ab8227) as a loading control.

### SARS‐CoV‐2‐infected cell blocks and lysates

To generate SARS‐CoV‐2‐infected/mock‐infected cell pellets for positive/negative control staining, African green monkey (Vero E6) cells (ATCC, American Type Culture Collection, Rockville, MD, USA) were incubated with SARS‐CoV‐2 D614G virus (hCoV‐19/England/IC19/2020 (EPI_ISL_475572)) diluted in Dulbecco's Modified Eagle's Medium (DMEM) at a multiplicity of infection (MOI) of 3 (or with plain media for mock‐infected cells) for 1 h at 37 °C, after which DMEM was removed and replaced with fresh medium supplemented with 10% foetal bovine serum (Labtech, Heathfield, UK, 1% nonessential amino acids and 1% penicillin–streptomycin; Gibco, Thermo Fisher Scientific). Cells were collected 48 h after infection or mock‐infection by scraping, washed in ice‐cold PBS twice, and fixed in formalin at room temperature for 24 h. Fixed cells were washed twice in PBS, centrifuged, and the pellet was processed using the Thermo Scientific Shandon Cytoblock system according to the manufacturer's instructions.

For protein lysate preparation, infections were performed as above but at an MOI of 0.1. Cells were collected by scraping 48 h postinfection, pelleted, and lysed in 500 μl ice‐cold RIPA buffer on ice for 30 min. Lysates were clarified by centrifugation at 4 °C for 30 min, then heated at 90 °C for 10 min in Laemmli buffer (BioRad, Hercules, CA, USA) with 10% β‐mercaptoethanol (Sigma‐Aldrich).

### Biosafety statement

All work performed at Imperial College London was approved by the local genetic manipulation safety committee of Imperial College London, St. Mary's Campus (centre number GM77), and the Health and Safety Executive of the United Kingdom, under reference CBA1.77.20.1.

### Animal models

Tissues from rhesus macaques and Syrian golden hamsters were obtained from experiments carried out at UKHSA Porton Down (Wiltshire, UK). Nonhuman primates (NHPs), specifically rhesus macaques (Indian origin) were inoculated via intranasal and intratracheal route with 5 × 10^6^ PFUs of SARS‐CoV‐2 (hCoV‐19/Australia/VIC01/2020) and culled at 4 or 7 days postchallenge [21]. Syrian golden hamsters were inoculated by the intranasal route with 5 × 10^4^ PFUs of the same SARS‐CoV‐2 isolate. Hamsters were culled at 2 days postchallenge. The infected status of macaques and hamsters was confirmed by RNAScope *in situ* hybridisation for S‐gene [21]. Samples from uninfected animals were obtained from the UKHSA Porton Down Pathology archive. All experimental work performed at UKHSA Porton Down was conducted under the authority of a UK Home Office approved project license (PDC57C033) that had been subject to local ethical review at UKHSA Porton Down by the Animal Welfare and Ethical Review Body (AWERB) and approved as required by the Home Office Animals (Scientific Procedures) Act 1986.

### Statistical analysis

All statistical analyses were performed using GraphPad Prism 8 (Graphpad Software, San Diego, CA, USA). Significance of disease duration between negative and positive postmortem cases was determined using Mann–Whitney *U* tests.

## Results

### Validation of anti‐NP monoclonal antibody

**Figure 1:**
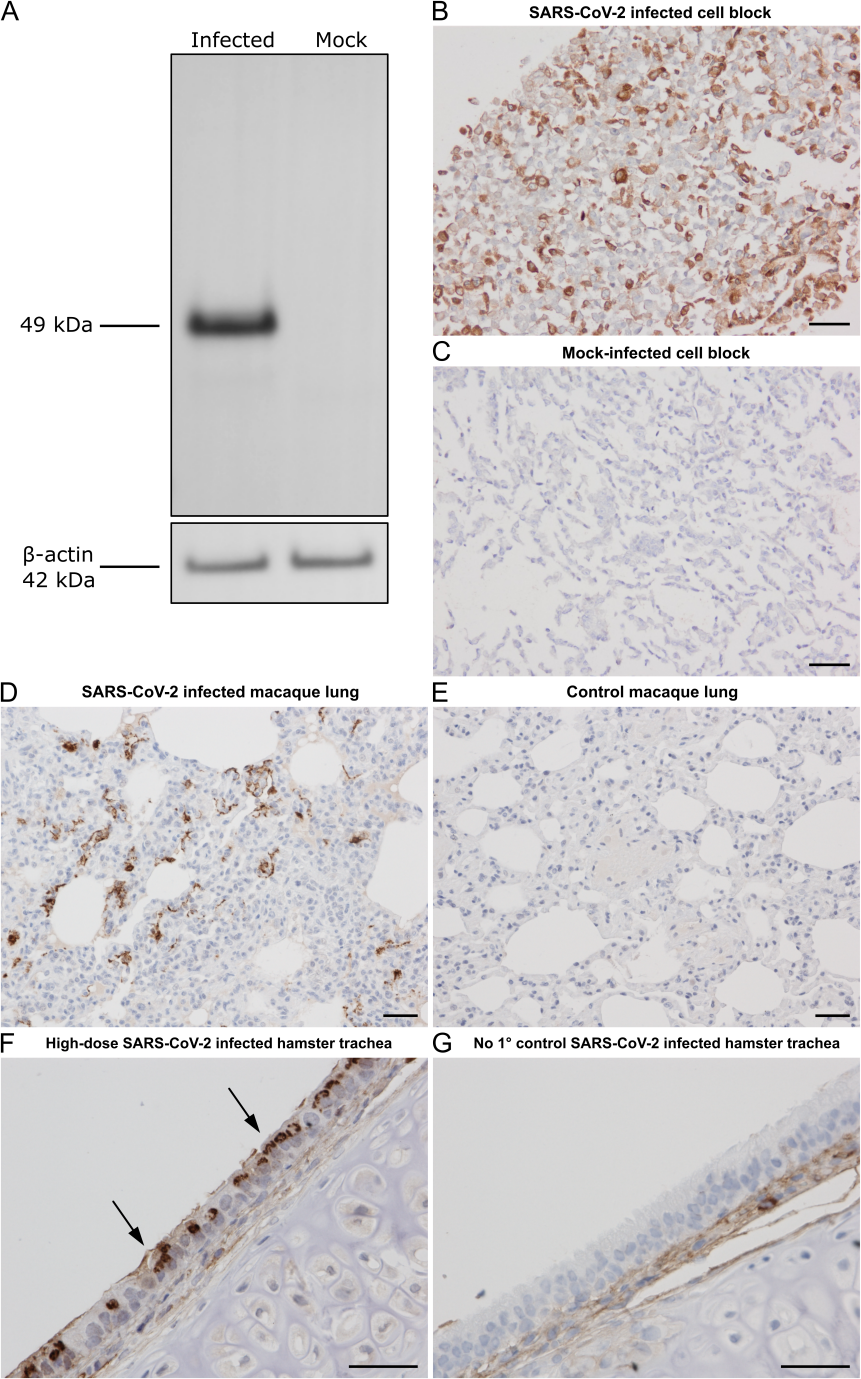
Validation of anti‐NP monoclonal antibody. (A) Western blot of anti‐NP monoclonal antibody using lysates of SARS‐CoV‐2‐infected and mock‐infected Vero E6 cells. Immunocytochemical (ICC) staining of (B) SARS‐CoV‐2‐infected Vero E6 cultured cells and (C) mock‐infected cells. IHC on the lungs of (D) SARS‐CoV‐2‐infected and (E) noninfected rhesus macaque models. (F,G) IHC of Syrian golden hamsters inoculated with a high dose of SARS‐CoV‐2. (F) Positive respiratory epithelium (arrows) in the trachea is demonstrated alongside (G) a no primary control of the same respiratory tract. The no primary antibody control allowed us to distinguish between specific SARS‐CoV‐2 NP immunoreactivity and nonspecific background staining, which was sometimes present when our staining protocol (optimised for human tissue) was used on hamster tissue. Scale bars = 50 μm.

Specificity of the anti‐NP monoclonal antibody was evaluated using western blotting and IHC. A single band of 49 kDa, the predicted molecular mass of the NP, was detected in lysates from SARS‐CoV‐2‐infected Vero E6 cells (Figure 1A, lane 1), whilst no signal was observed in lysates from mock‐transfected cells (Figure 1A, lane 2). By IHC, strong cytoplasmic immunoreactivity was present in SARS‐CoV‐2‐infected cells (Figure 1B), whilst no staining was observed in mock‐infected cells (Figure 1C). The specificity of the anti‐NP antibody for IHC was further validated in animal models of SARS‐CoV‐2. In the rhesus macaque model of SARS‐CoV‐2, there was strong positive cytoplasmic immunostaining within the lung parenchyma (Figure 1D) in contrast to noninfected rhesus macaques, where staining was not apparent (Figure 1E). In Syrian golden hamsters infected with SARS‐CoV‐2, similar strong immunoreactivity was observed in the respiratory tract and lung parenchyma (Figure 1F), but not in technical negative controls (Figure 1G).

### Postmortem cases: clinical characteristics and relationship of tissue SARS‐CoV‐2 NP presence to disease duration

We examined tissue from 16 postmortem cases from the peaks of the COVID‐19 pandemic in 2020 (see Table 1 for detailed clinical characteristics). The average age of patients was 64.8 years (range 22–97), with 12 of the 16 (75%) being male. Fourteen patients (88%) had significant comorbidities, of which hypertension and type 2 diabetes mellitus were the most common (38 and 31%, respectively). The average time from symptom onset to time of death was 21.1 days (range 8–46 days). The average postmortem interval was 7.5 days (range 1–15 days). In all 16 cases the underlying cause of death (part I of the medical certificate cause of death, MCCD) was attributed to SARS‐CoV‐2 infection and/or its sequelae (see Table 1 for detailed clinical characteristics).

Of the 10 cases that had tissue available for multisystem analysis (Table 1, PM1‐PM10), four showed SARS‐CoV‐2 NP by IHC (Table 2) and in all four, the viral presence was multisystemic. The average time from symptom onset to time of death was significantly shorter in the positive versus the negatively stained cases (mean = 10.3 days, SD = 3.3 days versus mean = 20.3 days, SD = 6.3 days, Mann–Whitney: U = 2, *p* = 0.0416), with all positive cases dying 15 days or earlier from symptom onset, whilst no positive staining was seen in patients with a longer disease courses.

**Table 2:** Summary of SARS‐CoV‐2 anti‐NP immunohistochemical organ localisation in postmortem COVID‐19 cases.

Organs and tissues	Cases									
	PM6	PM3	PM1	PM10**	PM4	PM2	PM7	PM8	PM9	PM5
Bladder	‐		P		‐	‐	‐	‐	‐	‐
Bone marrow	P	P	P	‐	‐			‐	‐	
Brain	E	E	E		‐	‐	‐	‐	‐	‐
Colon	E + P	E + P	P		‐	‐	‐	‐	‐	‐
Duodenum	P	P	P		‐	‐	‐	‐	‐	‐
Gallbladder	E	P	E + P		‐			‐	‐	‐
Heart	E	E		‐	‐	‐	‐	‐	‐	‐
Ileum	E + P	E	E + P		‐	‐	‐	‐	‐	‐
Kidney	P	P	P	P	‐	‐	‐	‐	‐	‐
Larynx	E + P	E + P	E + P		‐	‐				‐
Liver	E + P	E	E + P	E + P	‐	‐	‐	‐	‐	‐
Lung	E + P	P	P	P	‐	‐	‐	‐	‐	‐
Lymph node	E	‐	E			‐	‐	‐	‐	‐
Nasal epithelium	E + P	E + P	E + P						‐	
Oesophagus	E + P	E + P	E + P		‐	‐	‐	‐	‐	
Pancreas		P		‐	‐	‐	‐	‐		‐
Pharynx	E + P	P								
Sexual organs*	E + P	‐	P		‐			‐	‐	
Spleen	E	‐	E	‐	‐	‐	‐	‐	‐	‐
Stomach	P	‐	P		‐	‐	‐	‐	‐	‐
Thyroid			‐		‐	‐	‐	‐	‐	‐
Tongue	E + P	P	E + P		‐	‐	‐	‐	‐	‐
Trachea	E + P		E + P		‐	‐	‐	‐	‐	
Disease duration (days)	8	8	10	15	12	13	23	23	24	27

Key: (‐) = Negative for endothelial or parenchymal cells. (E) = Endothelial cells only. (P) = Parenchymal cells only. (E + P) = Endothelial and parenchymal cells.

* Prostate or ovary depending on patient sex.

** Postmortem percutaneous biopsy. Blank spaces indicate tissues that were not sampled.

### Respiratory system

**Figure 2:**
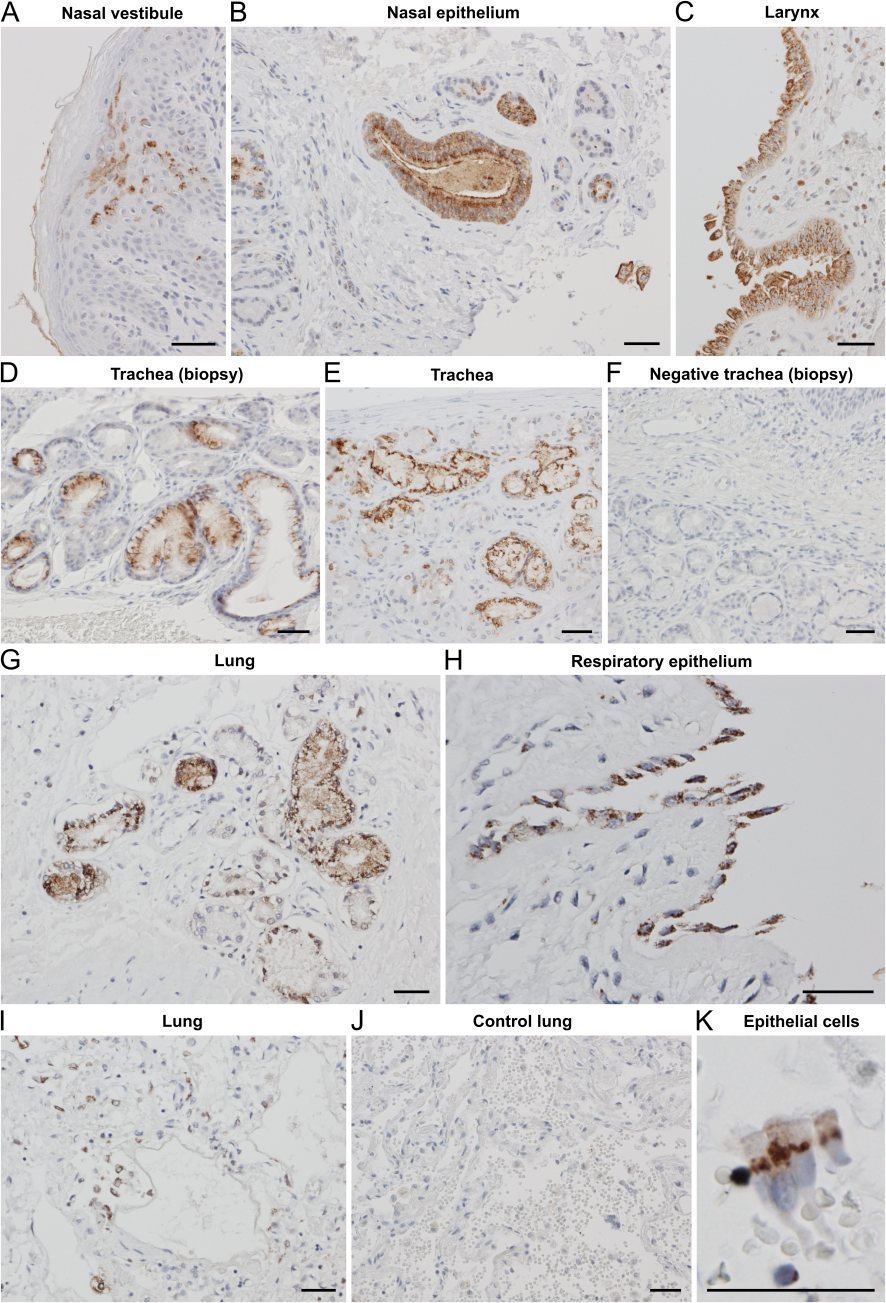
Cellular tropism of SARS‐CoV‐2 in the respiratory system. All images show IHC with anti‐NP antibody. (A) Focal staining in stratified squamous epithelium of nasal vestibule (PM1). (B) In nasal epithelium, serous and mucinous glands in the lamina propria are positive, as well as the surface epithelium, seen here in an invagination of the mucosa (PM1). (C) Viral presence in pseudostratified columnar epithelium in larynx (PM6). (D) Staining pattern of submucosal seromucinous glands in tracheal biopsy is similar to (E) positive postmortem trachea (TB7). (F) Negative tracheal biopsy for comparison (TB11). (G) Lung (PM6), with parabronchial seromucinous glands, as well as respiratory bronchial epithelium showing punctate pattern (H). (I) Widespread distribution of positive pneumocytes (PM6). (J) Pre‐COVID control lung is negative (C3). (K) Detail of ciliated respiratory epithelial cells showing cytoplasmic paranuclear and apical punctate pattern (PM3). Scale bars = 50 μm.

Tissue SARS‐CoV‐2 was visualised by IHC in the respiratory system of four out of ten cases with multisystem postmortem tissue available. Within the nasal cavities, scattered staining was apparent in stratified squamous epithelium of the vestibule (Figure 2A), as well as in serous and mucinous glands of the lamina propria and the surface nasal epithelium (Figure 2B). Seromucinous gland staining was commonly patchy, with strongly positive elements near negative ones. Within the larynx, pseudostratified columnar epithelial cells displayed strong staining, as did submucosal seromucous glands (Figure 2C). A similar pattern of seromucinous gland, epithelial, and endothelial cell staining was observed in the positive tracheal biopsies (Figure 2D) and postmortem tracheae (Figure 2E), with appropriate lack of staining shown in a PCR negative biopsy (Figure 2F). Staining was also apparent in pulmonary parabronchial seromucinous glands (Figure 2G). At the subcellular level, the staining pattern was predominantly an apical cytoplasmic or perinuclear punctuate pattern, a pattern particularly evident in surface epithelial cells (Figure 2H,K). Widespread positivity in alveolar pneumocytes was also observed (Figure 2I). As expected, there was no apparent anti‐NP positivity in SARS‐CoV‐2‐negative pre‐COVID control lungs (Figure 2J). Throughout the respiratory tract, hyaline cartilage was consistently negative for anti‐NP staining.

### Digestive system and myenteric plexus

**Figure 3:**
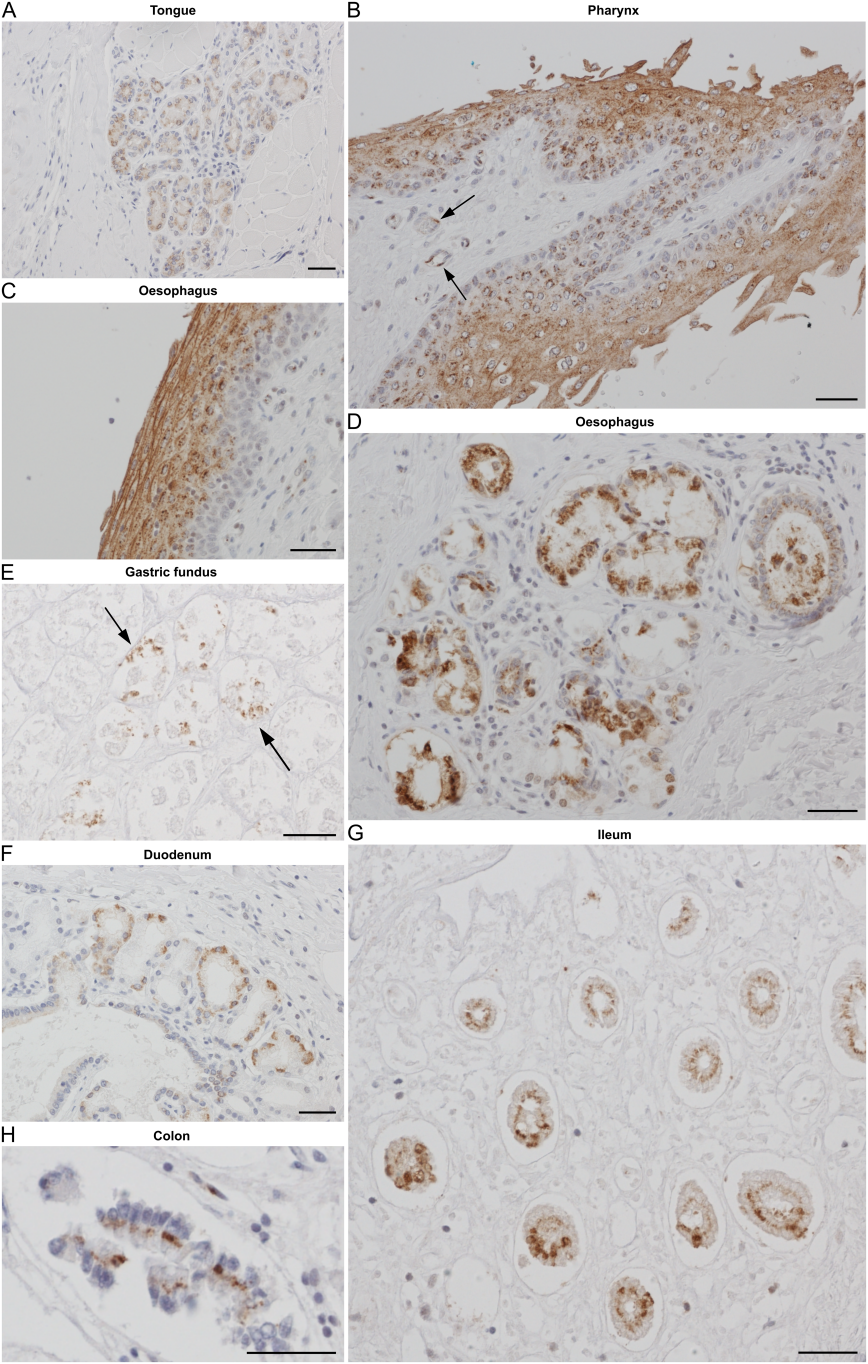
Cellular tropism of SARS‐CoV‐2 in the digestive system. All images show IHC with NP antibody. (A) Tongue accessory serous salivary glands (PM1). Stratified squamous epithelium of (B) pharynx (PM6) and (C) oesophagus (PM6) with the typical punctate pattern; arrows indicate endothelial labelling. (D) Oesophageal submucosal mucous glands (PM6). (E) Arrows show focal immunostaining of gastric glands (PM1). In the mucosa of the small intestine, positivity is mainly seen in the crypts and less so in the villi in (F) duodenum (PM1) and (G) ileum (PM6). (H) Positivity in small vesicle‐like punctate structures in the apical and paranuclear cytoplasm in the epithelium of a gland of the descending colon (PM6). Scale bars = 50 μm.

Moderate intensity staining was observed within the minor salivary glands and tongue epithelium as well as in endothelial cells (Figure 3A). Definite taste buds were not identified. Strong positive staining was apparent in the pharyngeal and oesophageal epithelium (Figure 3B,C), with scattered staining of underlying endothelial cells (Figure 3B, arrow) and submucosal glands (Figure 3D). Weaker scattered positive staining was apparent in parietal cells of the gastric fundus (Figure 3E). In the small intestine, mucosal staining was preferentially located to the epithelial cells within glandular crypts (Figure 3F,G), whilst in the colon focal scattered positive staining was noted in colonic glands (Figure 3H).

**Figure 4:**
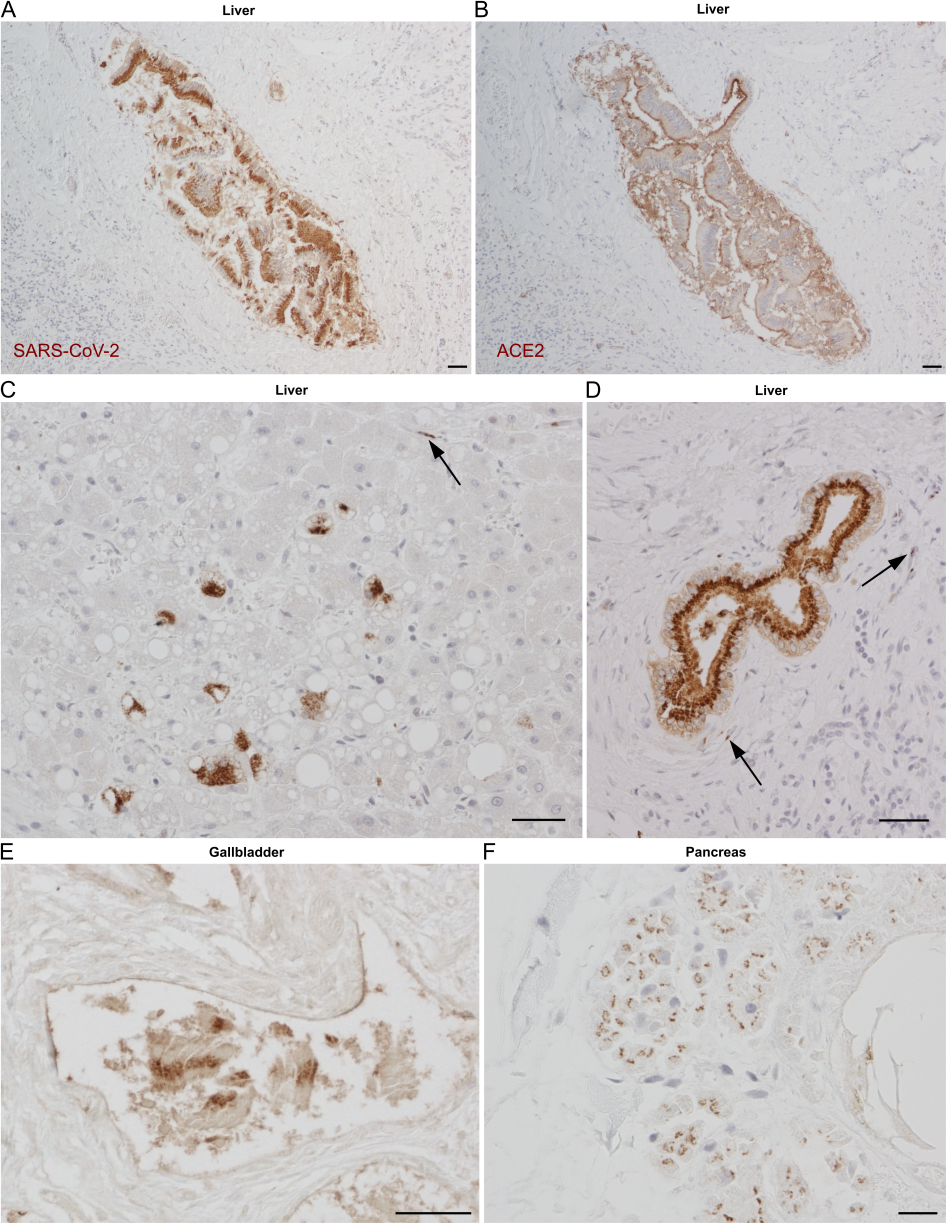
Cellular tropism of SARS‐CoV‐2 in the liver and other digestive organs. Consecutive sections immunostained for (A) SARS‐CoV‐2 entry receptor ACE2 or (B) SARS‐CoV‐2 NP demonstrate matching staining in the hepatic bile duct (PM1). (C) Liver parenchymal NP staining was limited to occasional steatotic hepatocytes (PM1); arrow shows endothelial staining. (D) Hepatic bile duct with typical punctate apical cytoplasmic epithelial staining; arrows indicate adjacent endothelial positivity (PM1). (E) Columnar epithelium of the gallbladder showing perinuclear NP (PM3). (F) Pancreatic acini with widespread, dotted NP positivity (PM3). Scale bars = 50 μm.

SARS‐CoV‐2 NP was present in cholangiocytes of the hepatic bile ducts (Figure 4A), correlating with the localisation of anti‐ACE2 immunostaining (Figure 4B), with ACE2 and SARS‐CoV‐2 situated predominantly on the apical end of the cells. Strong diffuse positive staining was seen throughout the hepatobiliary system in the portal tracts (Figure 4B,D), with focal staining in adjacent hepatocytes in areas with steatosis (Figure 4C). Endothelial viral presence was also observed in the proximity of positive hepatocytes (Figure 4C, arrow) and bile ducts (Figure 4D, arrows). Strong diffuse positive staining was also apparent in columnar epithelial cells of the gallbladder (Figure 4E) and scattered weak staining in the pancreatic acini (Figure 4F). Strong ACE2 staining was observed in epithelial cells from bile ducts, gall bladder, and pancreatic ducts in noninfected nonhuman primate tissues (data not shown).

**Figure 5:**
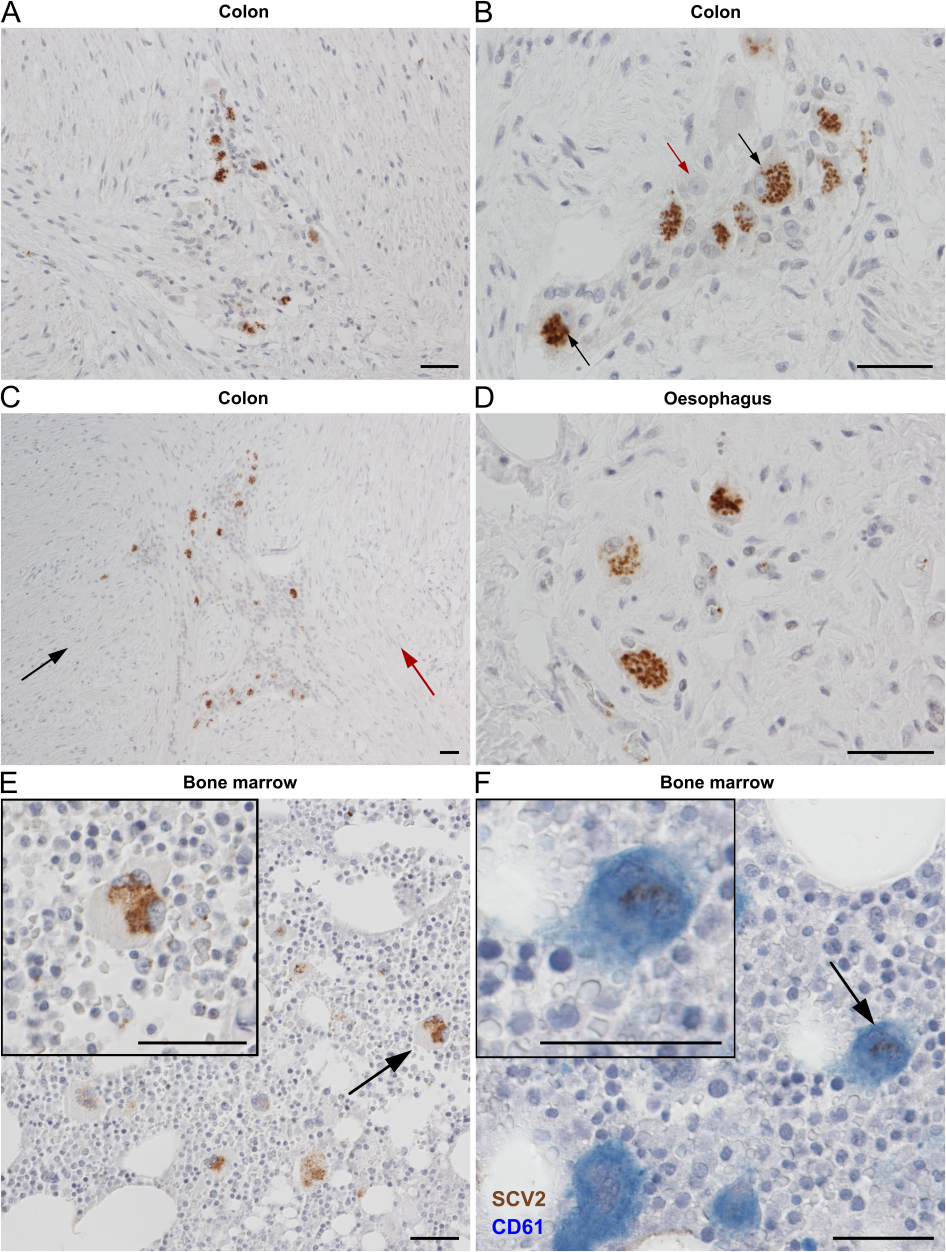
Presence of SARS‐CoV‐2 in neurons of the myenteric plexus and in megakaryocytes in the bone marrow. (A) NP immunostaining of ganglia of the myenteric plexus (PM6). (B) Higher magnification reveals punctate staining within neuronal soma (arrows). Red arrow shows a negative neuron. (C) Overview of extensive positivity within the myenteric plexus. Black arrow points to concentric muscle layer, red arrow points to longitudinal muscle layer. (D) NP staining in neurons of oesophageal myenteric ganglia (PM6). (E) NP staining in megakaryocytes (arrow) with their distinct lobular nuclei. The inset shows the typical paranuclear cytoplasmic punctate pattern (PM10). (F) SARS‐CoV‐2 in megakaryocytes (see arrow and inset, PM3) is confirmed by colocalisation of NP (brown) with the megakaryocyte marker CD61 (blue). Scale bars = 50 μm.

Conspicuous strong staining was identified within neurons of the myenteric plexus, typically located between the smooth muscle layers in the colon (Figure 5A–C). Paranuclear, vesicle‐like punctate staining in the soma of some, but not all, enteric ganglion cells (Figure 5B, —arrows). Oesophageal myenteric plexus also showed viral positivity within ganglia (Figure 5D). Supportive glial cells intercalated between the neurons in the myenteric ganglia were unstained (Figure 5B). The submucosal plexus was not sufficiently represented in the samples available for analysis.

Alpha‐synuclein aggregation in Parkinson's disease (PD) is thought to begin in the neurons of the enteric nervous system [22], where cholinergic neurons are particularly susceptible to alpha‐synuclein pathology [23, 24]. Consequently, we used immunostaining for ChAT (for cholinergic neurons) and nNOS (for nitrergic neurons) to assess any preference of SARS‐CoV‐2 for a specific neuronal subtype. NP was visualised within both cholinergic and nitrergic neurons (supplementary material, Figure S1). No SARS‐CoV‐2 staining was seen in the digestive system, including the myenteric plexus, in pre‐COVID postmortem controls (supplementary material, Figure S2).

### Haematopoietic system and megakaryocytes

Within the bone marrow, there was SARS‐CoV‐2 NP present within megakaryocytes (Figure 5E), confirmed by colocalisation with CD61 (Figure 5F). Similar to other cells, viral protein showed a punctate pattern concentrated in the paranuclear cytoplasmic region. Scattered staining in endothelial cells was seen in spleen and lymph node (supplementary material, Figure S3A,B). No SARS‐CoV‐2 staining was seen in the haematopoietic system in pre‐COVID postmortem controls (supplementary material, Figure S2).

### Genitourinary system

**Figure 6:**
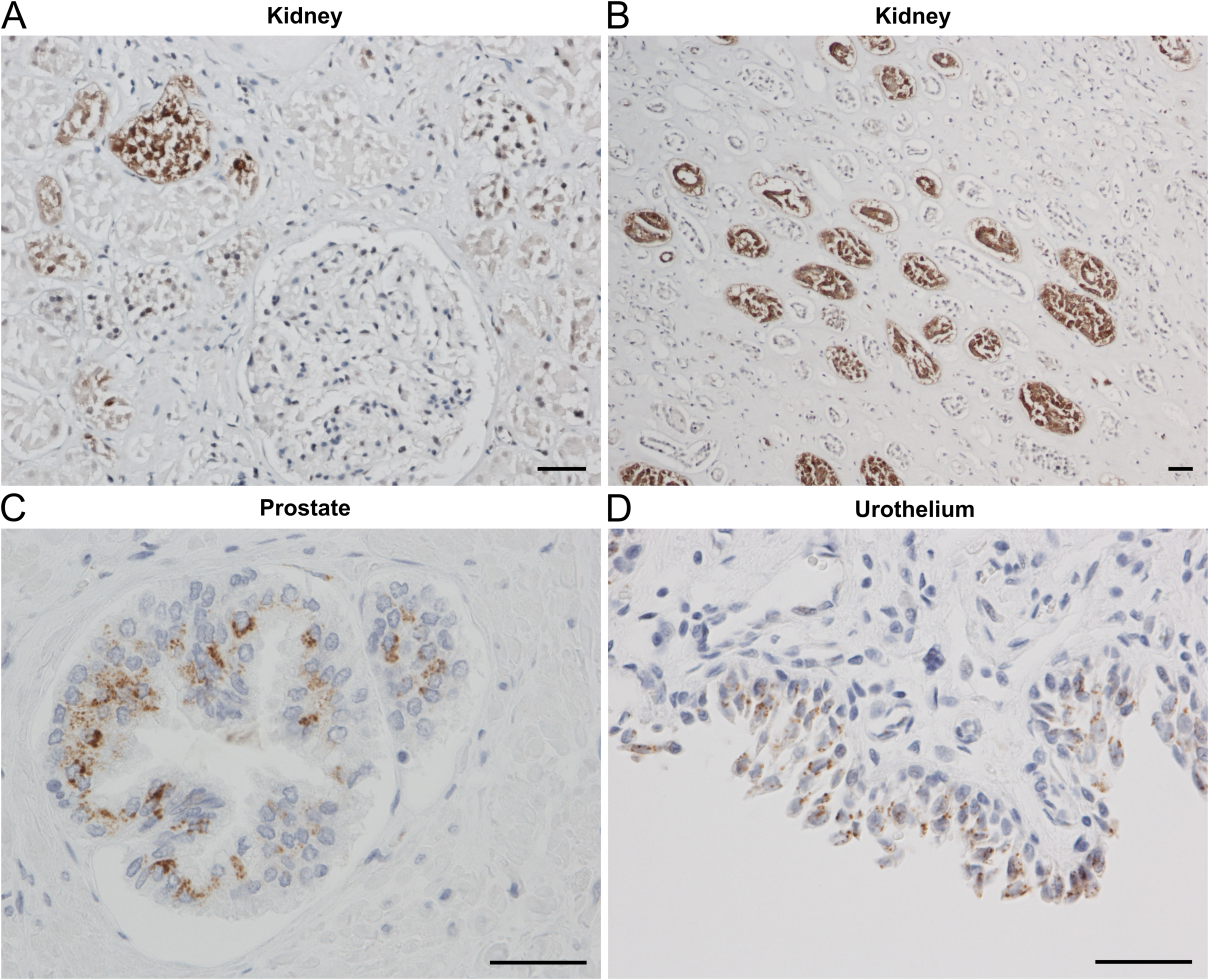
Cellular tropism of SARS‐CoV‐2 in the genitourinary system. Images show **i**mmunostaining for NP. (A) SARS‐CoV‐2 is seen in the epithelium of convoluted tubules in the renal cortex, but glomeruli are largely negative. (B) Viral presence in collecting tubules in the renal medulla (PM3). (C) Prostatic glands and (D) urothelium (PM1) demonstrate the typical paranuclear cytoplasmic punctate pattern. Scale bars = 50 μm.

Within the renal cortex, strong positive staining for SARS‐CoV‐2 NP localised to the epithelium of convoluted tubules (Figure 6A), but glomeruli were largely negative except for occasional endothelial glomerular labelling. In the renal medulla, a striking viral presence was identified in collecting tubules (Figure 6B). Scattered positive staining was noted in prostatic acinar cells in urothelial cells in the bladder (Figure 6C,D).

### Central nervous system

SARS‐CoV‐2 NP stained only endothelial cells, with no localisation to neuronal or glial cells (supplementary material, Figure S4). When present, endothelial staining was widespread in all brain regions sampled in postmortem cases, which included frontal cortex (supplementary material, Figure S4A), medulla, cerebellum (supplementary material, Figure S4B), pons, and olfactory bulb (Table 3). Furthermore, in the full‐body postmortem cases, positivity in brain endothelia was restricted to cases exhibiting multiorgan tissue positivity (PM1, 3 and 6). Notably, in the olfactory bulb, a postulated entry site for the virus into the brain, SARS‐CoV‐2 was restricted to endothelial cells and neurons were negative (supplementary material, Figure S4C). A proportion of cases in our extended brain cohort (PM11‐16, BB1), which also included brain‐only postmortems and a brain biopsy, displayed widespread endothelial staining. However, there was no apparent relationship between the endothelial labelling and the presence of neurological symptoms, perivascular or parenchymal inflammation, or brain infarction. In the positively stained case showing fatal brainstem encephalitis (PM12), staining was seen both in vessels with perivascular lymphocytic infiltrates (supplementary material, Figure S4G) and in uninflamed vessels (supplementary material, Figure S4D). In contrast, the other case that displayed brainstem encephalitis (PM4) [15] and the case displaying acute disseminated encephalomyelitis (ADEM) in the context of COVID‐19 (BB1), displayed negative immunostaining for SARS‐CoV‐2 NP (supplementary material, Figure S4F). In cases with brain infarction, we found brain endothelial labelling in one case, including in vessels with perivascular lymphocytic infiltrates near the infarction (supplementary material, Figure S4E; PM15) and in uninflamed vessels distant from the infarction, but not in other cases (PM16, PM5). Staining was not apparent within ependymal cells (supplementary material, Figure S4H). No SARS‐CoV‐2 staining was seen in the brain in pre‐COVID postmortem controls (supplementary material, Figure S2).

**Table 3:** Summary of SARS‐CoV‐2 NP localisation in the brain of COVID‐19 cases.

Brain regions	Cases														
	PM1	PM2	PM3	PM4	PM5	PM6	PM8	PM9	PM11	PM12	PM13	PM14	PM15	PM16	BB1
Frontal Cortex	E	‐	E	‐	‐	E	‐	‐	‐	E	E−/+		E	‐	‐
Medulla			E	‐	‐				‐	E	‐	E			
Cerebellum	E	‐	E	‐		E	‐	‐	‐	E	E−/+		E	‐	
Pons			E	‐	‐				‐	E			E		
Olfactory bulb	E	‐	E	‐	‐	E	‐	‐							

Only endothelial cells were observed to show positivity in the brain, but when present, it appeared throughout the brain. As such, E represents positive staining in endothelial cells and E−/+ indicates weak positivity in endothelial cells. No definite parenchymal SARS‐CoV‐2 was seen in any case.

### Endothelium

Endothelial staining, when present, was not only restricted to the brain regions studied, but was multisystemic across several organs (supplementary material, Figure S3). Nevertheless, the distribution of viral staining was variable, with some organs showing broad staining across all vessels (e.g. brain) and others showing focal staining only. In the heart, positivity was observed within endothelial cells but not within any other cell type, including myocytes (supplementary material, Figure S3F). No SARS‐CoV‐2 staining was seen in the endothelia in pre‐COVID postmortem controls (supplementary material, Figure S2).

### Tracheal biopsies

We also examined tracheal biopsies of 15 individuals who had been admitted to intensive care and required tracheostomy (see Table 4 for detailed clinical characteristics). Their average age was 55 years (range 33–68) and consisted of eight females (53%) and seven males (47%). Seven of the tracheal biopsies showed the presence of SARS‐CoV‐2 NP by IHC (Table 4, TB1‐TB7 and Figure 2D). In five of these (TB1‐TB4, TB7), SARS‐CoV‐2 was confirmed by RT‐PCR nasopharyngeal swab prior to tracheal biopsy. The two remaining cases had persistently negative RT‐PCR SARS‐CoV‐2 nasopharyngeal swabs prior to biopsy (Table 4, TB5 and TB6). Nonetheless, participant TB5 had a respiratory picture which was entirely in keeping with COVID‐19, both clinically and radiologically. Participant TB6 did not have clinical features of COVID‐19, but required a prolonged admission to intensive care due to unrelated medical issues and may have been infected during this period (the admission was in Winter 2020; a period with high infection rates in the UK).

**Table 4:** Clinical characteristics of COVID‐19 cases who underwent tracheal biopsy.

Case	Age	Sex	Reason for admission to intensive care	Date of intubation	Date of tracheostomy	Nasopharyngeal swab results	Anti‐NP immunohistochemistry result
TB1	48	Female	COVID‐19 respiratory failure	31 March 2020	10 April 2020	Positive (31 March 2020, 1 April 2020) then negative (5 May 2020)	Positive
TB2	57	Female	COVID‐19 respiratory failure	28 March 2020	11 April 2020	Positive (23 March 2020, 24 March 2020) then negative (14 April 2020)	Positive
TB3	59	Male	COVID‐19 respiratory failure	30 March 2020	13 April 2020	Positive (26 March 2020)	Positive
TB4	48	Male	COVID‐19 respiratory failure	7 April 2020	20 April 2020	Positive (5 April 2020) then negative (6 May 2020)	Positive
TB5	63	Male	COVID‐19 respiratory failure	21 June 2020	23 July 2020	Negative (9 swabs performed between 18 June 2020 and 31 August 2020)	Positive (weak)
TB6	43	Female	Erythema multiforme of unclear precipitant, pontine stroke, and thrombocytosis	9 January 2020	4 February 2020	Negative (21 swabs performed between 31 December 2020 and 22 March 2020)	Positive (strong)
TB7	53	Male	COVID‐19 respiratory failure	9 January 2021	27 January 2021	Initially positive (5 January 2021). Showed mixed swabs with a negative on 22 January 2021 and subsequent negatives but positive swabs until 22 February 2021.	Positive
TB8	56	Male	COVID‐19 respiratory failure	4 April 2020	18 April 2020	Initially positive (3 April 2020) then negative (6 May 2020)	Negative
TB9	33	Female	COVID‐19 respiratory failure	3 March 2020	9 April 2020	Initially negative (26 March 2020 and 28 March 2020) then positive (28 March 2020)	Negative
TB10	55	Male	Major burns	15 August 2020	10 September 2020	Negative (11 swabs performed between 23 August 2020 and 17 October 2020)	Negative
TB11	57	Female	New presentation of myasthenia gravis	15 January 2020	22 January 2020	Negative (12 swabs performed between 13 January 2020 and 15 March 2020)	Negative
TB12	64	Female	COVID‐19 respiratory failure	10 January 2021	1 February 2021	Initially positive (3 January 21, 18 January 21) then negative (25 January 21)	Negative
TB13	68	Male	COVID‐19 respiratory failure	6 January 2021	27 January 2021	Initially positive (6 January 2021, 18 January 2021) then indeterminate 22 January 2021. Negative 29 January 2021.	Negative
TB14	68	Female	COVID‐19 respiratory failure	18 January 2021	1 February 2021	Initially positive (18 January 2021–8 February 2021) then negative (15 February 2021)	Negative
TB15	56	Female	Intracerebral bleed	25 January 2021	8 February 2021	Initially positive (25 January 2021, 1 February 2021) then negative (8 February 2021)	Negative

No definite SARS‐CoV‐2 NP was detected in the remaining eight tracheal biopsies by IHC (Figure 2F), and the agreement between negative IHC and nasopharyngeal RT‐PCR results was complete (Table 4, participants TB8‐TB15). Two of these cases (TB8 and TB9) had a clinical course compatible with COVID‐19, but they both had inconsistent SARS‐CoV‐2 nasopharyngeal swab results, with a documented negative swab result 18 days after and 12 days before, respectively, undergoing tracheal biopsy. Four cases (TB12‐TB15) initially presented with positive swab results but, by the time of tracheostomy, their swab results were negative. The remaining two cases (participants TB10 and TB11) were admitted to intensive care for reasons unrelated to COVID‐19 and had documented swab‐negativity repeatedly before and after tracheostomy, and hence were included as negative controls.

## Discussion

Predominantly a respiratory disease, it has been logical for COVID‐19 histopathological studies to focus on the respiratory system. Despite this, there is value in examining the histopathological correlates of SARS‐COV‐2 infection in extrapulmonary organs and tissues, given the increasing spectrum of extrapulmonary symptoms reported. Here, we performed a comprehensive multisystem organ analysis of SARS‐CoV‐2 cellular tropism in tissues from 36 patients with PCR‐confirmed SARS‐CoV‐2 status using anti‐NP IHC, yielding two key findings. First, we report the novel finding of SARS‐CoV‐2 NP within neurons of the myenteric plexus but not the brain. Our results provide evidence for peripheral neuroinvasion in the digestive tract, but critically, not in the CNS. Furthermore, we have demonstrated viral NP within platelet‐producing megakaryocytes, a finding that provides insight into the well‐characterised thrombotic sequelae of COVID‐19.

It had been previously speculated that, due to the expression of ACE2 in neurons of the myenteric plexus, these cells are possible targets of SARS‐CoV‐2 infection [2], a hypothesis that we corroborate here for the first time. The long‐term consequences of our findings in the myenteric plexus are yet to be established. PD is a neurodegenerative disorder thought to result from the prion‐like spread of amyloidogenic alpha‐synuclein causing catastrophic motor impairment and other symptoms, such as dementia, as the spread of misfolded protein progresses throughout the brain [25]. However, it is not known if the initial seeding of the misfolded protein conformation is purely a stochastic event or ignited by an internal or external factor, with the exception of 5–10% of cases in which genetic mutations have been shown to impact alpha‐synuclein biology [26]. The brain pathology of PD initiates in the olfactory bulb and medulla from where it propagates in a stereotypical pattern [11], and this is anticipated by accumulation of misfolded alpha‐synuclein in the ENS. This pattern of pathology progression explains anosmia and gastrointestinal (GI) symptoms such as constipation and nausea [27], appearing early in PD. It has been proposed that an environmental agent reaches the brain from the nose through the olfactory nerves or from the gut through the ENS *via* retrograde axonal transport along the vagus nerve reaching the medulla [12, 28].

Viral infection of myenteric neurons, as implied by our study, could in theory be one such environmental factor. Historically, links between viral infection and Parkinsonism were seen following the 1918 influenza–encephalitis lethargica pandemic [29]. During the COVID‐19 pandemic, there have been reports of concurrent neurological complications and encephalitis [20], however, there is currently only one report of postviral Parkinsonism development [30]. Diversity of genetic background, comorbidities, and previous infections all impact susceptibility and likely may only have neurodegenerative ramifications in a subset of people. As we begin to recover from the pandemic, we should nevertheless still be conscious of COVID‐19 when assessing future neurological cases.

On the other hand, the extent of SARS‐CoV‐2's direct invasion of the CNS is uncertain. Throughout the pandemic, neurological symptoms such as headache, ageusia, and anosmia were commonly reported, and the involvement of both the central and peripheral nervous system was hypothesised [31]. Our brain cohort of SARS‐CoV‐2‐infected patients includes patients with clinical and pathological neurological disease, including brainstem encephalitis and cases with no neurological involvement. However, positivity within the brain appeared to be limited to endothelial cells. Crucially, despite observing viral NP in the neurons of the myenteric plexus, we found the origin of the vagus nerve in the medulla oblongata to be devoid of neuronal SARS‐CoV‐2 NP (supplementary material, Figure S5). This suggests that, in the event of any neurodegenerative disease developing after SARS‐CoV‐2 infection, consideration should be given to the spread of misfolded protein or other pathophysiological alterations triggered by the virus in ENS neurons, rather than the direct entry of the virus into CNS cells. Previous reports about the possibility of viral protein or RNA in the CNS are conflicting [8, 14, 16, 32, 33]. One hypothesis postulates that systemic vascular changes are likely behind reported neurological symptoms, as an indirect consequence of lung‐derived hypoxia rather than direct viral damage. Additionally, endothelial cell targeting and injury are also considered to be key elements in COVID‐19 pathophysiology that have been frequently alluded to [34].

COVID‐19 has been established as a prothrombotic disease characterised by coagulopathy, thrombosis, and platelet activation [35, 36]. Our results parallel this with findings of platelet‐producing megakaryocytes colocalising with the viral NP within the bone marrow. Several publications have also reported the presence of circulating megakaryocytes in multiple organs, including lung, heart, kidney, liver, and brain [14, 37, 38, 39]. One study demonstrated that SARS‐CoV‐2 infection can promote procoagulant changes in platelet gene expression [40]. Paradoxically, despite confirming viral presence, the authors also ascertained that megakaryocytes and platelets lack ACE2, by which they suggested an independent route of infection. A recent preprint report that infected calprotectin‐expressing megakaryocytes additionally express the ACE2 receptor and TMPRSS2 [41]. Even more intriguing, that study also provided evidence of megakaryocytes harbouring infected pro‐platelets. Together with our own observations, it is probable that entry occurs by several mechanisms. The principal molecular route of SARS‐CoV‐2 entry into megakaryocytes and platelets is therefore still unresolved.

Our results imply appreciable involvement of the GI tract and related digestive organs in COVID‐19, correlating with the high prevalence of symptoms such as diarrhoea, abdominal pain, anorexia, and vomiting in COVID‐19 symptomatology [42]. Of note, the viral cell tropism in the digestive tract overall mirrors that of the expression profile of ACE2 in cell types such as enterocytes [43]. Preexisting chronic liver diseases, such as steatohepatitis and cirrhosis, have been commonly associated with severe cases of COVID‐19, with 2–11% of patients having liver comorbidities [44, 45]. With four of ten patients displaying liver disease, this is also a common finding within our own full‐body postmortem cohort. In addition to endothelial cells, three cases (PM1, PM6, and PM10) exhibited a striking viral NP presence within parenchymal tissues, including bile duct cholangiocytes, as well as hepatocytes. Studies of the ACE2 receptor in normal liver asserts low expression in hepatocytes; however, mouse models and human samples suggest chronic liver injury may contribute to ACE2 receptor upregulation [43, 46]. Cholangiocytes, on the other hand, purportedly have high levels of ACE2, which may explain the strong viral protein detection seen in this study.

We also observed, predictably, a diffuse viral presence throughout the respiratory system. Consistent with other reports, detection of viral NP in the lungs appears to be limited to seromucinous glands, alveolar pneumocytes, and respiratory epithelium [47]. As previously described, all the lungs in the PM1‐10 case series present with DAD, a histopathological aberration commonly affiliated with acute respiratory distress syndrome (ARDS) [8]. However, our immunohistochemical analysis revealed parenchymal positivity in only a subset of patients (four of ten). As NP has been canonised as a proxy for replicating SARS‐CoV‐2, this suggests that only a few patients in our cohort died with active pulmonary infection [48]. Considering the disease duration of these cases, it is likely that our findings capture the early phase of ARDS in which viral activity persists [49, 50, 51]. Similar observations have been made in other SARS‐CoV‐2 *in situ* studies, as well as in SARS‐CoV, where viral replication in the lungs is constrained to the early postinfective period (~2 weeks) [47, 52, 53].

Interestingly, we found that parenchymal SARS‐CoV‐2 presence in the lungs was associated in all cases with an extrapulmonary viral presence, and that this was also constrained to this ~2‐week period. Our data, with a universal endothelial presence in these cases, suggests that extrapulmonary dissemination could occur *via* haematogenous spread. This is supported by increased SARS‐CoV‐2 RNA load in the blood (RNAemia) to be associated with disease severity in COVID‐19 patients [54, 55] and by multiorgan damage to be related to high RNAemia [56], although other studies disagree [57]. In the case of GI viral presence, a faecal‐oral route of transmission is possible [58], supported by detection of viral RNA in the faeces of COVID‐19 patients, even after testing negative in respiratory tissues in a subset of patients [59]. Our data also suggest that beyond this ~2‐week period, COVID‐19 multiorgan complications may be the result of persistence of viral tissue damage and of pathophysiological cascades ignited by the viral infection, including inflammatory activation, rather than continued viral infection. Given that our postmortem samples are from patients who died during the initial first COVID‐19 wave in the UK, our study cannot comment on any vaccine‐related inflammatory processes.

A strength of this study is the strict validation performed on our immunostaining protocol. Consistently throughout our study we identified a characteristic immunostaining pattern to be indicative of SARS‐CoV‐2. After gaining entry into host cells, viral RNA and replicative protein components form a reticulovesicular network by reorganising the host endoplasmic reticulum [60]. We have identified that, in most cell types, genuine SARS‐CoV‐2 positive stain displays a cytoplasmic apical or paranuclear punctate pattern reflecting the viral cycle. In addition, the clinical relevance of this antibody is best illustrated by our tracheal biopsy series in which we used our protocol to show clinicopathological correlations. Out of 15 biopsies, the vast majority of SARS‐CoV‐2 IHC results were consistent with the timeline of nasopharyngeal swab results, as well as overall symptom presentation. The outliers included one patient who persistently had negative swabs yet showed very strong IHC positivity. As the tracheostomy occurred a considerable time after the last swab, it is likely this patient had been infected during their prolonged admission to the ICU, subsequent to tracheostomy and tracheal biopsy.

Our study has limitations. Despite comprehensive sampling, our full‐body cohort is of limited size, with some tissues being subjected to autolysis as well as sampling bias. This makes it difficult to speculate on any enteric neuronal loss or cytopathic damage seen in patients who died later in their disease course, although we did find evidence for at least a proportion of the neurons in the myenteric plexus surviving into the postreplicative phase (supplementary material, Figure S6). Second, we cannot completely exclude the possibility of cross‐reaction with SARS‐CoV‐1, although, to our knowledge, this virus is not currently in circulation in our community. Contextualising the patients analysed in this study (e.g. date and location of death), it is highly unlikely that we have misidentified another betacoronavirus, particularly as the cases had confirmed SARS‐CoV‐2 PCR positivity. Additionally, when considering nucleoprotein homology, the closest sequence (bat coronavirus nucleoprotein) only reveals 75% alignment (supplementary material, Figure S7).

Overall, our investigation has demonstrated the multisystemic tropism of SARS‐CoV‐2. Concordant with other histopathological publications, we have shown extrapulmonary involvement, with an emphasis on the digestive system, in severe cases. Most significantly, we provide evidence of neuroinvasiveness in the ENS but not the CNS. We have also identified megakaryocytes as potential targets of SARS‐CoV‐2. Further investigations in gene expression profiling related to these findings will help determine any long‐term implications.

## Author contributions statement

SGR carried out experiments, performed analysis, and cowrote the article. MJ collected and analysed data and cowrote the article. MOJ collected information and cowrote the article. PW collected and analysed data and revised the article. CR developed experimental resources and revised the article. BH, EVA, JW, ZJ, SB, UP, KA, MT, CT, SAS, SG, DG, MC, WB and MO collected experimental resources and revised the article. OS, IF, NQ, FS and MS carried out experiments and revised the article. JAA conceived the study, obtained funding, performed data collection, analysis, and interpretation, collected experimental resources, and cowrote the article. All authors had final approval of the submitted and published versions.

## Supporting information


**Figure S1.** Presence of SARS‐CoV‐2 in cholinergic and nitrergic neurons of the myenteric plexus
**Figure S2.** Absence of anti‐SARS‐CoV‐2 immunostaining in pre‐COVID controls
**Figure S3.** SARS‐CoV‐2 tropism to the endothelium is multisystemic
**Figure S4.** Cellular tropism of SARS‐CoV‐2 in the brain
**Figure S5.** Absence of anti‐SARS‐CoV‐2 immunostaining in dorsal vagal nucleus
**Figure S6.** Immunohistochemistry for anti‐SARS‐CoV‐2 in the myenteric plexus of PM4
**Figure S7.** Sequence homology between anti‐SARS‐CoV‐2 antibody target peptides
**Table S1.** Clinical characteristics of pre‐COVID control cases
