# Supplementary material for: Multisystem screening reveals SARS‐CoV‐2 in neurons of the myenteric plexus and in megakaryocytes
Source: J Pathol. 2022 Mar 31;257(2):198–217. doi: 10.1002/path.5878 (PMC9325073; doi:10.1002/path.5878)
Supplement: Supplementary file 1 — Figure S1. Presence of SARS‐CoV‐2 in cholinergic and nitrergic neurons of the myenteric plexus Figure S2. Absence of anti‐SARS‐CoV‐2 immunostaining in pre‐COVID controls Figure S3. SARS‐CoV‐2 tropism to the endothelium is multisystemic Figure S4. Cellular tropism of SARS‐CoV‐2 in the brain Figure S5. Absence of anti‐SARS‐CoV‐2 immunostaining in dorsal vagal nucleus Figure S6. Immunohistochemistry for anti‐SARS‐CoV‐2 in the myenteric plexus of PM4 Figure S7. Sequence homology between anti‐SARS‐CoV‐2 antibody target peptides Table S1. Clinical characteristics of pre‐COVID control cases [file PATH-257-198-s001.docx]

**Multisystem screening reveals SARS-CoV-2 in neurons of the myenteric plexus and in megakaryocytes**

S Gray-Rodriguez *et al*. *J Pathol* DOI: 10.1002/path.5878

**Supplementary Figures S1–S7**

**Supplementary Table S1**


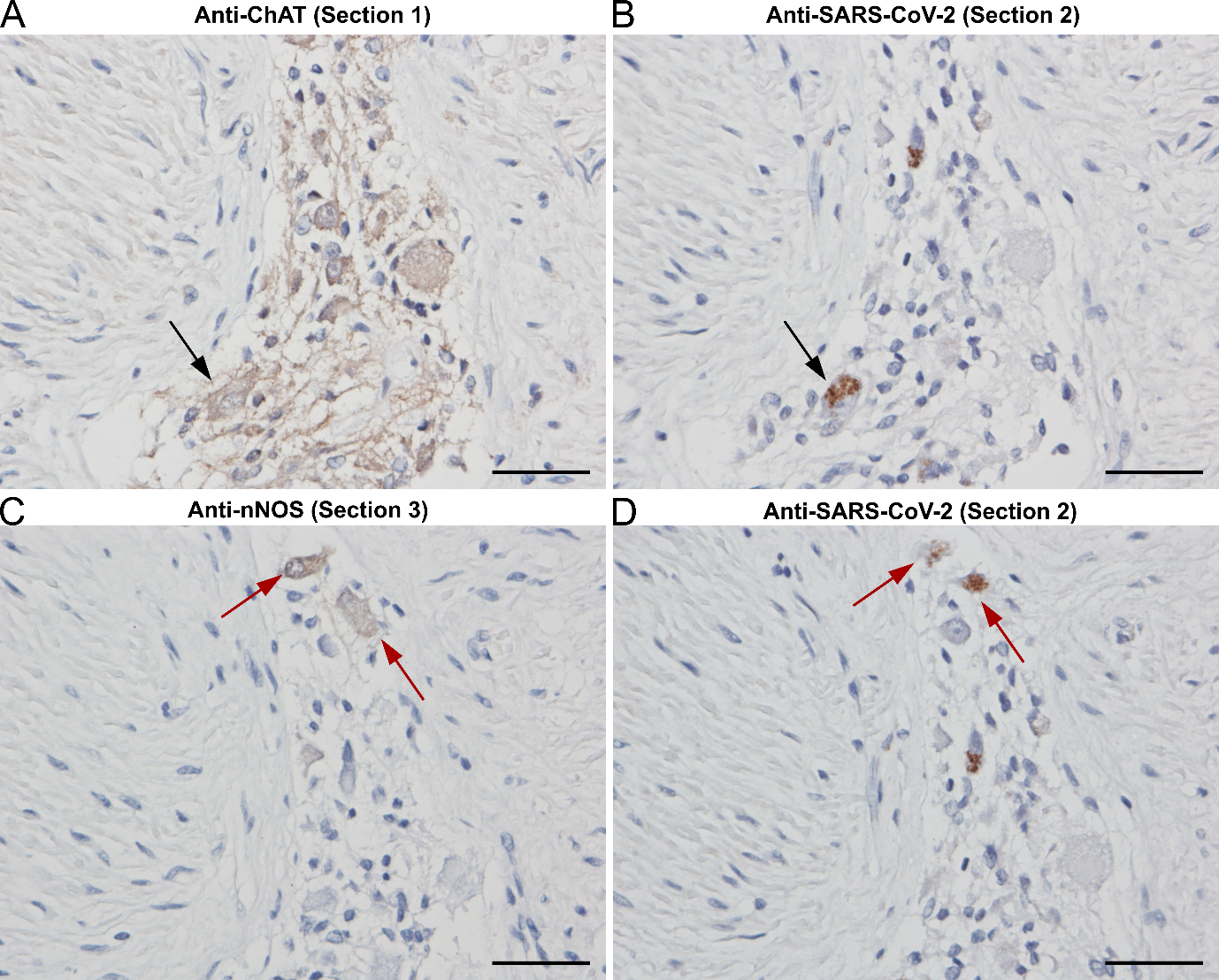


**Figure S1. Presence of SARS-CoV-2 in cholinergic and nitrergic neurons of the myenteric plexus.**

(A and B) Consecutive sections of myenteric plexus (PM6) were immunostained with anti-ChAT (A) and anti-SARS-CoV-2 NP (B). Arrows in A and B identify the same cholinergic neuron also showing viral presence. (C and D) Consecutive sections to those in A and B were immunostained with anti-nNOS (C) and anti-SARS-CoV-2 NP (D). Arrows in C and D identify two nitrergic neurons also showing viral presence. All scale bars shown represent 50μm.


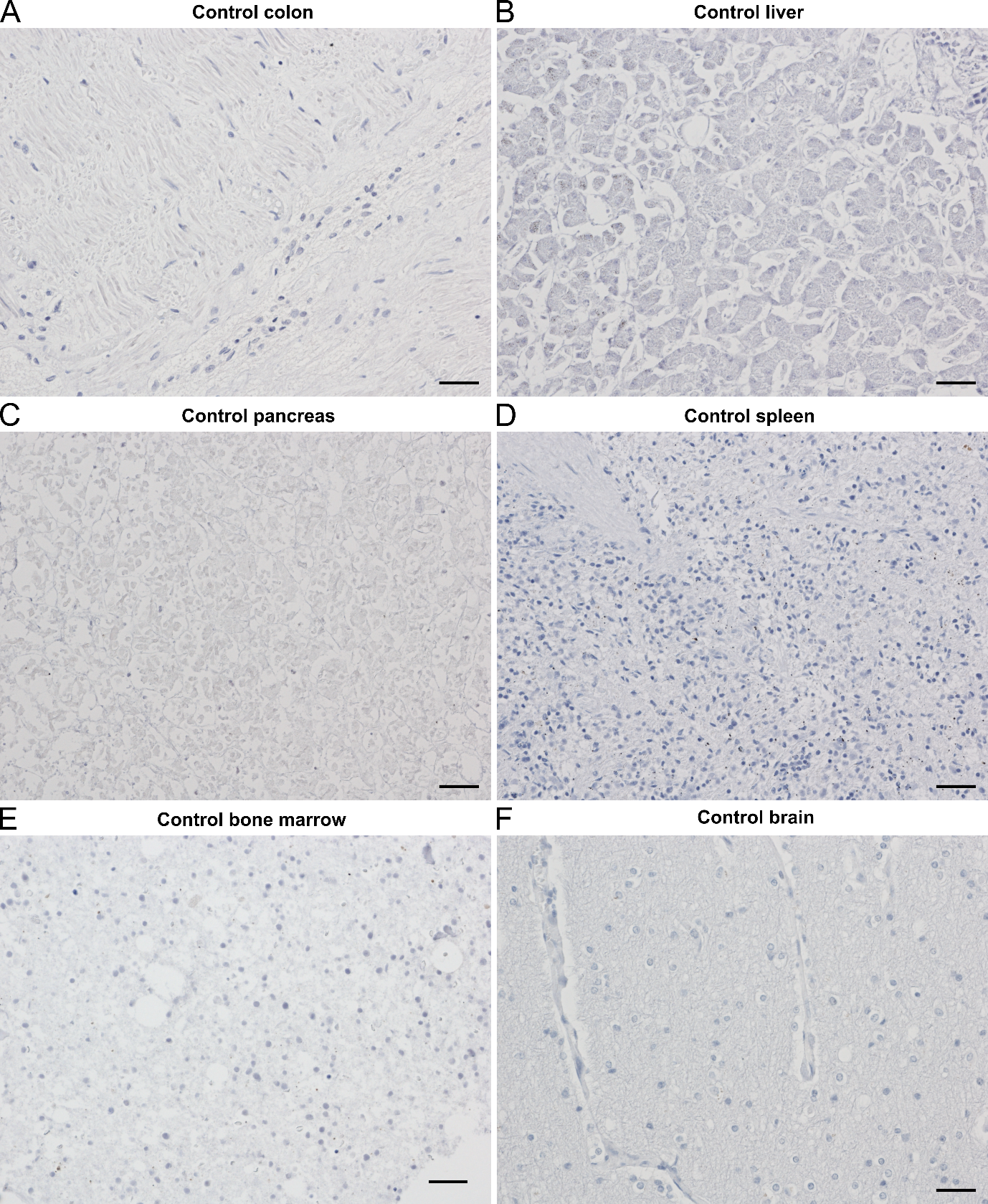


**Figure S2. Absence of anti-SARS-CoV-2 immunostaining in pre-COVID controls.**

Representative images of control cases showing lack of staining in (A) the colonic myenteric plexus (C1), (B) hepatocytes (C4), (C) pancreas (C4), (D) spleen (C4), (E) bone marrow (C2) and (F) cortex (C4). Refer to Supplementary Table 1 for clinical details. All scale bars shown represent 50μm.

**
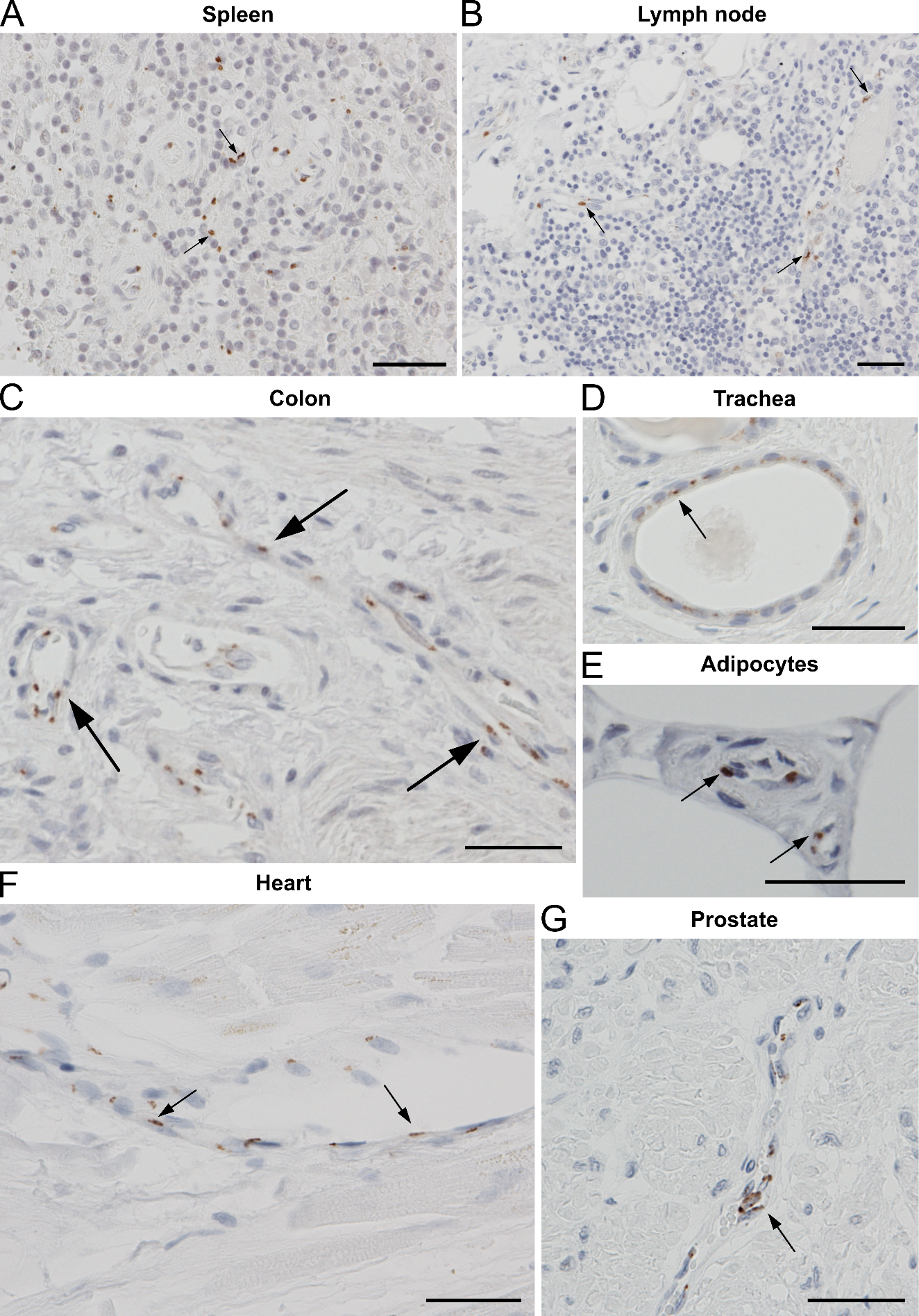
**

**Figure S3. SARS-CoV-2 tropism to the endothelium is multisystemic.**

(A) Red pulp of spleen (PM1) with arrows showing endothelial labelling in several vessels. (B) SARS-CoV-2 NP protein is also detected in lymph node endothelial cells (PM1). SARS-CoV-2 endothelial invasion using IHC for the NP antibody is also found within endothelial cells in (C) descending colon (PM6), (D) trachea (TB17), (E) adipose tissue (PM1), (F) left ventricle of the heart (PM3) and (G) prostate (PM6). All scale bars shown represent 50μm.

**
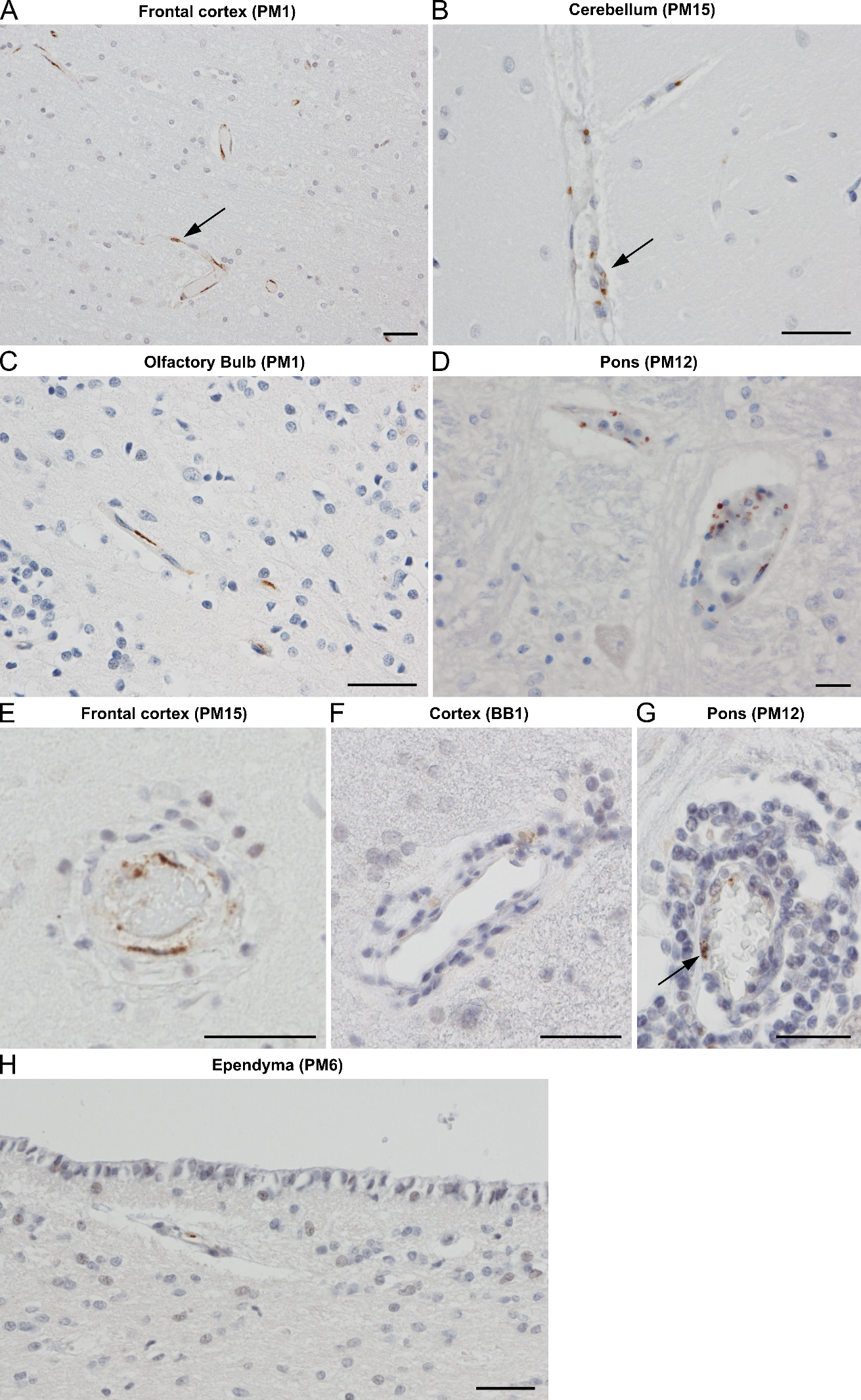
**

**Figure S4. Cellular tropism of SARS-CoV-2 in the brain.**

(A) Representative image of frontal cortex (PM1). Endothelial labelling (arrow) is widespread in all cases and regions in which there is multisystem tissue SARS-CoV-2 presence. (B) Cerebellum (PM15) with arrow showing viral presence in endothelial cells. (C) Olfactory bulb demonstrating positivity in vessels but not within neurons (PM1). No clear-cut relationship of endothelial SARS-CoV-2 and perivascular inflammation is seen. (D) Endothelial labelling in uninflamed vessels in the lower pons of PM12. (E) Positive staining with mild perivascular inflammation in frontal cortex (PM15). (F) Comparatively, in patient with necrotising encephalitis (BB1), staining is negative (some perivascular haemosiderin deposits are present). (G) Distinct positivity is observed in endothelial cells surrounded by an inflammatory infiltrate in lower pons (PM12). (H) Positive staining was not observed within ependymal cells (PM6). All scale bars shown represent 50μm.

**Figure S5. Absence of anti-SARS-CoV-2 immunostaining in dorsal vagal nucleus.**

Neurons of the dorsal vagal nucleus demonstrate a lack of staining in medulla oblongata of PM6. Same case showed positivity in neurons of the myenteric plexus. Scale bar shown represents 50μm.


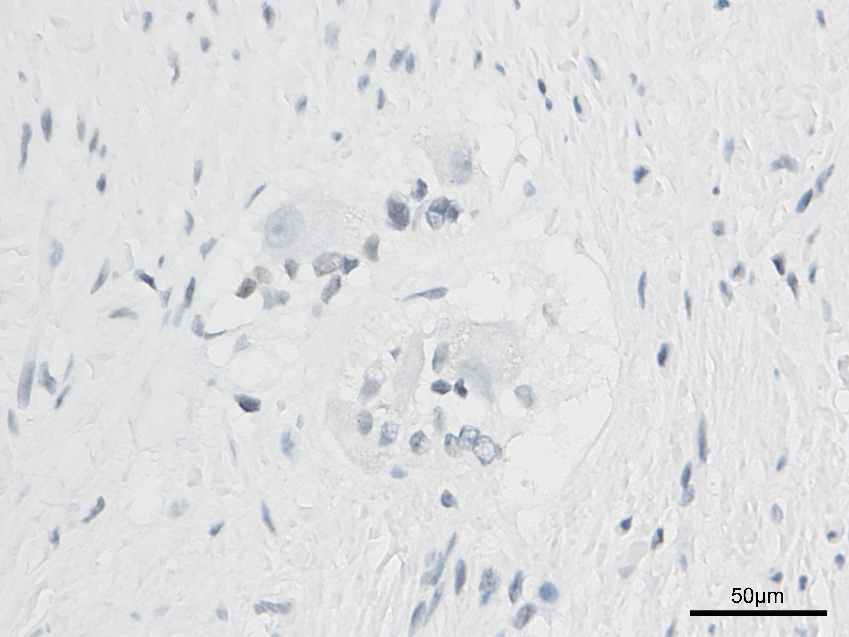


**Figure S6. Immunohistochemistry for anti-SARS-CoV-2 in the myenteric plexus of PM4.**

In cases with longer disease course, there was no immunoreactivity for anti-SARS-CoV-2 NP in neurons (arrow) of the myenteric plexus.


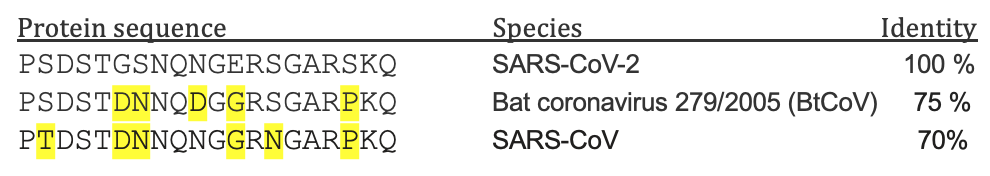


**Figure S7. Sequence homology between anti-SARS-CoV-2 antibody target peptides**

Alignments of target peptide sequence used in generation of mouse anti-SARS-CoV-2 Nucleoprotein monoclonal antibody (clone 4B21). Sequence alignment performed using <https://www.uniprot.org/>. Differences in amino acid sequence highlighted.

**Table S1. Clinical characteristics of pre-COVID control cases**

Details of post-mortem cases (pre-COVID pandemic) referenced as negative controls for anti-SARS-CoV-2 immunostaining.

| **Case** | **Age (years)** | **Sex** | **Death date** | **Clinical history** |
| --- | --- | --- | --- | --- |
| C1 | 68 | M | August 2019 | Cause of death:  1a: Acute myeloid leukaemia |
| C2 | 40 | F | July 2018 | DAD, fibrinous pericarditis, enterococcus infection  Cause of death:  1a: Ischaemic small bowel  1b: Acute graft versus host disease  1c: Bone marrow transplant for CMML-2 |
| C3 | 87 | M | April 2019 | History of myocardial infarction, ischaemic heart disease  Cause of death:  1a: Bradycardia  1b: Ischaemic heart disease |
| C4 | 59 | M | July 2017 | PCP, lung disease, DAD, history of HIV  Cause of death:  1a: Multi Organ Failure  1b: PCP Pneumonia |

DAD = Diffuse alveolar damage. CMML-2 = Chronic Myelomonocytic Leukemia-2. PCP = Pneumocystis pneumonia.
